# The role of cognitive function in predicting metabolic risk in schizophrenia: a multi-model comparison incorporating clinical features

**DOI:** 10.3389/fpsyt.2025.1724238

**Published:** 2026-01-12

**Authors:** Rui Li, Xuan Ren, Tingyun Jiang, Jiawen Huo, Junjiao Ping, Shuyi Zhu, Aoxiang Luo

**Affiliations:** 1 School of Nursing, Guangdong Pharmaceutical University, Guangzhou, Guangdong, China; 2 Department of Psychiatry, The Third People’s Hospital of Zhongshan City, Zhongshan, Guangdong, China

**Keywords:** schizophrenia, metabolic syndrome, cognition, machine learning, random forest, support vector machine, risk stratification

## Abstract

**Background:**

Patients with schizophrenia frequently exhibit metabolic abnormalities that are closely associated with cognitive impairment. However, clinically applicable risk-stratification tools based on concise and generalizable indicators remain limited. This study evaluated the predictive value of cognitive and clinical features for metabolic risk stratification and compared the discriminative performance of traditional statistical and machine-learning models.

**Methods:**

In this cross-sectional study, 213 patients with schizophrenia who received treatment at Zhongshan Third People’s Hospital between September 2024 and September 2025 were enrolled according to the Diagnostic and Statistical Manual of Mental Disorders, Fifth Edition (DSM-5). Based on the diagnostic criteria for metabolic syndrome in the Chinese Guideline for the Prevention and Treatment of Type 2 Diabetes (2017 edition), patients were categorized into three groups: High-risk, Critical, and MS. General clinical data, symptom ratings, and cognitive assessments (Chinese version of the MATRICS Consensus Cognitive Battery, MCCB) were collected. Features were selected using the Boruta algorithm and screened for multicollinearity, followed by the construction of multinomial logistic regression, random forest (RF), extreme gradient boosting (XGBoost), and support vector machine (SVM) models; class imbalance was addressed using SMOTE.

**Results:**

Years of education, processing speed, verbal learning, visual learning, and reasoning/problem solving demonstrated stable and independent predictive contributions across models. Age, age at onset, and negative symptoms were also retained during feature selection. The RF model achieved the best overall discriminative performance (macro-average AUC = 0.789; Macro-F1 = 0.603), whereas the SVM model showed superior performance in identifying minority classes (balanced accuracy = 0.725; Macro-F1 = 0.625). These results remained consistent after controlling for clinical symptoms and general demographic variables.

**Conclusions:**

Modeling based on concise clinical and cognitive indicators can effectively achieve metabolic risk stratification in patients with schizophrenia. Rather than relying on a single algorithm, combining the complementary strengths of RF and SVM and selecting models according to specific clinical needs and data characteristics may improve the identification of high-risk individuals and support proactive intervention and management.

## 1 Introduction

Schizophrenia is a severe and chronic psychiatric disorder characterized not only by persistent psychotic symptoms and impaired social functioning but also by pervasive cognitive deficits. Cognitive impairment is considered a core feature of schizophrenia, involving multiple domains such as reasoning and problem solving, processing speed, verbal learning, attention, and executive function (1, 2).Accumulating evidence suggests that cognitive deficits are closely related to patients’ social recovery and long-term outcomes (3, 4). However, current pharmacological treatments have shown limited efficacy in improving cognitive performance (5, 6). Therefore, identifying and intervening on risk factors associated with cognitive dysfunction remain key priorities in both clinical practice and research.

Meanwhile, the prevalence of metabolic syndrome (MetS) is markedly higher among patients with schizophrenia than in the general population, reaching 30%–50% (7, 8). Growing evidence indicates that metabolic abnormalities not only increase the risk of cardiovascular disease and premature mortality but may also interact with cognitive dysfunction in schizophrenia (9, 10). Several studies have reported that metabolic risk factors—such as obesity, hyperglycemia, and dyslipidemia—are associated with multidimensional cognitive impairments, including deficits in attention, memory, and executive function (11, 12). However, other studies have failed to identify independent associations between metabolic abnormalities and cognition, suggesting that this relationship may be moderated by disease stage or other covariates (13). Moreover, many prior studies on metabolism and cognition did not systematically include multiple cognitive domains (14), which may have contributed to inconsistencies in the existing findings. Nonetheless, biological processes such as chronic inflammation, insulin resistance, and oxidative stress appear to overlap in their involvement in both metabolic abnormalities and cognitive impairment, providing partial mechanistic clues for their potential association (10, 15, 16). Building on this evidence, we hypothesized that patients with different levels of metabolic risk may exhibit distinct patterns across specific cognitive domains, with key domains such as processing speed and verbal learning potentially showing greater sensitivity.

Furthermore, treating MetS as a binary variable may not fully capture the spectrum of metabolic risk characteristics (17), whereas refined stratification could better identify high-risk individuals and guide clinical interventions (18).In this study, we adopted a risk-stratification approach based on the number of metabolic abnormality components, as recommended in previous guidelines. This method was chosen to reflect a graded spectrum of metabolic risk from mild to severe and to emphasize the clinical relevance of identifying the intermediate stage. Methodologically, previous studies have relied predominantly on traditional logistic regression. Although machine-learning techniques have demonstrated clear predictive advantages in psychiatric research (19–21), their application to metabolic-risk prediction has largely focused on imaging or biomarker data, limiting their clinical practicality (22).Moreover, clinical characteristics and cognitive function reflect different dimensions of disease burden in schizophrenia, providing complementary information and thereby offering a theoretical basis for integrating both domains into risk prediction. In contrast to computational psychiatry studies that rely on high-cost, multimodal data, the conventional clinical indicators and cognitive assessments used in this study are far more accessible and operationally feasible, helping to bridge the “translation gap” between predictive modeling and everyday clinical practice.

Building on this context, we hypothesized that cognitive indicators in schizophrenia would be associated with metabolic risk stratification, and that different cognitive domains would contribute differentially to the discrimination of risk categories. In addition, given the potential advantages of machine-learning methods in handling nonlinear structures and high-dimensional features, we further hypothesized that predictive performance would vary across modeling strategies, with certain machine-learning models outperforming traditional multinomial logistic regression.

## 2 Methods

### 2.1 Participants

This cross-sectional study enrolled patients with schizophrenia who received treatment at Zhongshan Third People’s Hospital between September 2024 and September 2025. The study protocol was approved by the Ethics Committee of Zhongshan Third People’s Hospital (approval No.SSYLL-KY-20240905). All participants (or their legal guardians) provided written informed consent after receiving a full explanation of the study procedures.

Inclusion criteria: (i) diagnosis of schizophrenia according to the Diagnostic and Statistical Manual of Mental Disorders, Fifth Edition (DSM-5) (23); (ii) age between 18 and 60 years; (iii) clinically stable and able to complete all assessments; and (iv) written informed consent obtained from the participant or guardian.

All diagnoses were independently confirmed by two qualified attending psychiatrists based on clinical history and structured interviews. Any discrepancies were resolved through consultation with a senior psychiatrist to determine the final diagnosis.

Exclusion criteria: (i) comorbid severe psychiatric or neurological disorders; (ii) severe cardiac, hepatic, or renal dysfunction, or pregnancy or lactation; (iii) serious physical illness or substance dependence; and (iv) intellectual disability or incomplete clinical or assessment data.

In total, 213 patients met the inclusion criteria. It is important to note that no unified standard currently exists for sample size estimation in multi-class machine-learning prediction models. Methodological literature instead emphasizes the use of robust validation strategies to enhance the reliability of model performance when sample sizes are limited (24).Accordingly, this study employed several robustness procedures during model development to mitigate potential biases arising from sample-size constraints. The detailed modeling and validation steps are presented in Section 2.8, “Machine Learning Modeling and Validation.”

### 2.2 Outcome

The diagnosis of metabolic syndrome was determined according to the Chinese Guideline for the Prevention and Treatment of Type 2 Diabetes (2017 edition) (25).In accordance with this guideline, metabolic syndrome is identified when three or more of the following five components are present (1): Central obesity: waist circumference ≥90 cm in men or ≥85 cm in women (2). Hyperglycemia: fasting plasma glucose ≥6.1 mmol/L; or 2-h plasma glucose ≥7.8 mmol/L during an oral glucose tolerance test; or a previous diagnosis of diabetes (3). Hypertension: blood pressure ≥130/85 mmHg, or current treatment with antihypertensive medication (4). Hypertriglyceridemia: fasting triglyceride level ≥1.70 mmol/L (5). Low HDL-C: fasting HDL-C level <1.04 mmol/L.

Metabolic risk stratification followed the Expert Consensus on the Management of Metabolic Syndrome in Schizophrenia (26).This consensus defines individuals with schizophrenia as a high-risk population for metabolic syndrome as a whole. Accordingly, patients who do not meet any metabolic-syndrome components are still classified as the High-risk group. Those meeting 1–2 components are categorized as the Critical group, representing an intermediate metabolic-risk level between the High-risk and MS groups. Individuals meeting ≥3 components are classified as the MS group. These three groups served as the outcome categories in subsequent multi-class prediction modeling.

### 2.3 Predictors

The candidate predictors covered multiple domains, including demographic characteristics, clinical symptoms, and cognitive function. Demographic and clinical information included Age, Sex, Age at Onset, Duration, Education, Marital Status, Living Arrangement, Upbringing, Smoking, Alcohol use, Family History of Psychiatric Disorders, Family History of Metabolic Disorders, Antipsychotic Combination, and Antipsychotic Risk. Clinical symptoms were assessed using the Positive and Negative Syndrome Scale (PANSS), and the P subscore, N subscore, G subscore, and Total score were recorded (27).

Cognitive function was assessed using the Chinese version of the MATRICS Consensus Cognitive Battery (MCCB), which covers six core cognitive domains: processing speed, attention/vigilance, working memory, verbal learning, visual learning, and reasoning/problem solving. The processing speed domain includes three subtests: Trail Making Test–A, BACS Symbol Coding, and Category Fluency.

Raw scores from each subtest were converted into standardized T-scores (mean = 50, SD = 10) using the official MCCB Computer Scoring Program (Version 3.16.2; University of California & SIStat, 2016) and age-corrected normative data. These T-scores were used in subsequent statistical and modeling analyses to ensure comparability and standardization across cognitive domains.

All assessments were administered by trained raters during the clinically stable phase, following standardized procedures to ensure scoring consistency and reliability. During assessment, raters were not informed of participants’ metabolic status or their risk stratification based on metabolic indicators. The diagnosis of metabolic syndrome and the corresponding risk classification were determined independently after data collection, based on laboratory findings and relevant guidelines. Although it is not feasible in routine clinical settings to completely blind assessors to patients’ physical appearance (e.g., body habitus), the use of unified and standardized assessment procedures helps reduce potential evaluation bias.

To develop an early risk-stratification model centered on cognitive characteristics and to reduce the risk of outcome-related redundancy or over-adjustment, metabolic indicators that were isomorphic with the outcome or positioned downstream in its causal pathway were intentionally excluded. Likewise, lifestyle factors and medication exposure variables—whose distributions often vary substantially across clinical sites—were not incorporated. This approach allowed the model to focus on identifying metabolic risk using readily accessible clinical information, including demographic, symptomatic, and cognitive features, without relying on laboratory tests or behavioral data.

### 2.4 Data preprocessing

During data cleaning, an integrity check was first performed on the raw dataset. Missing values were handled using complete-case analysis to avoid potential bias introduced by data imputation. Qualitative variables were uniformly converted into categorical factors and, when necessary, expanded into dummy variables during model construction. The distribution of continuous variables was examined using the Shapiro–Wilk test.

Before entering the machine learning models, data were standardized according to the specific characteristics of each algorithm. For the support vector machine (SVM), which relies on distance-based metrics, all continuous variables were standardized prior to model fitting to eliminate the influence of differences in measurement scales. In contrast, tree-based algorithms, such as the random forest (RF) and extreme gradient boosting (XGBoost) models, were not standardized, as these algorithms are inherently robust to variable scaling (28, 29).

### 2.5 Descriptive statistics and group comparisons

To examine baseline differences among the metabolic risk groups, all candidate variables were first subjected to descriptive statistical analysis. Categorical variables were expressed as frequencies and percentages, and group comparisons were performed using the Pearson chi-square test or Fisher’s exact test, depending on the expected cell counts. Continuous variables were summarized as medians and interquartile ranges (IQRs), and intergroup differences were assessed using the Kruskal–Wallis rank-sum test. When the overall group difference reached statistical significance, Dunn’s *post hoc* test with Holm correction was applied for pairwise comparisons. All statistical tests were two-tailed, and the significance level was set at α = 0.05.

### 2.6 Feature selection and multicollinearity diagnostics

Before model construction, a two-stage variable selection procedure was conducted to eliminate multicollinearity and redundant predictors, combining the Boruta algorithm and variance inflation factor (VIF) filtering.

First, the Boruta algorithm—a random forest–based all-relevant feature selection method (30)—was applied to rank the importance of all candidate variables. Features were classified as confirmed important by comparing their importance scores with those of corresponding shadow features.

Subsequently, variables retained by Boruta were subjected to multicollinearity diagnostics. Complete collinearity was first screened using the alias function (31), followed by the computation of variance inflation factors (VIFs) (32, 33). The final set of predictors included in the modeling process was determined based on the combined results of Boruta feature selection and VIF-based collinearity diagnostics.

### 2.7 Multinomial logistic regression

In the traditional statistical analysis, a multinomial logistic regression model (34, 35)was employed to examine the associations between candidate predictors and metabolic risk stratification. The outcome variable was categorical with three levels—High-risk, Critical, and MS—using the High-risk group as the reference category. Model parameters were estimated using the maximum likelihood method, and results were presented as odds ratios (ORs) with corresponding 95% confidence intervals (95% CI) and Wald test *p*-values.

In this model, continuous variables were included in their original measurement scales, while categorical variables were converted into dummy variables before being entered into the regression equation.

### 2.8 Machine learning modeling and validation

To further evaluate the discriminative performance of different algorithms in predicting metabolic risk stratification, three commonly used machine learning models were developed and compared: random forest (RF), extreme gradient boosting (XGBoost), and support vector machine (SVM) with a radial basis function (RBF) kernel. These models were selected for the following reasons: the RF algorithm can handle high-dimensional features and provides robust interpretability through its built-in feature importance measures (28, 36);XGBoost, a widely adopted gradient-boosting approach, has demonstrated superior discriminative performance in medical prediction tasks (37, 38); and SVM, which is based on the principle of margin maximization, is particularly suitable for high-dimensional small-sample data and offers advantages in recognizing minority classes (21, 39).

Given the relatively modest sample size in this study—and the increased susceptibility of machine-learning performance estimates to bias under small-sample conditions—we employed a validation framework centered on cross-validation, combined with a systematic hyperparameter-tuning procedure, to enhance the robustness of the modeling results (24).

To address class imbalance in the outcome variable, the Synthetic Minority Oversampling Technique (SMOTE) was applied independently within the training folds of each model to balance class distributions, whereas the outer validation folds and test sets retained the original class distribution to avoid information leakage and ensure the independence and reproducibility of cross-validation results (40).In addition, to prevent overfitting and obtain reliable estimates of generalization performance, all SMOTE-based models were trained and evaluated using a nested 5-fold (outer) × 5-fold (inner) cross-validation scheme, with the inner loops used for hyperparameter optimization and the outer loops for performance evaluation (41). In each outer fold, 80% of the data were used for training and 20% for validation.

Building on the data preparation described above, we first constructed a random forest (RF) model using the original dataset and evaluated its performance through 10-fold cross-validation with out-of-fold predictions, in which 90% of the data were used for training and the remaining 10% for validation in each fold. Under the SMOTE-oversampled data, the RF model was further optimized within the nested cross-validation framework, tuning hyperparameters such as the number of trees and maximum tree depth. Similarly, the XGBoost model was fine-tuned through nested cross-validation, with a search space covering common hyperparameters including nrounds, max_depth, and eta (42). The SVM model, employing an RBF kernel, constructed the classification boundary by optimizing the penalty parameter (C) and kernel coefficient (γ) within the inner cross-validation loop, using the macro-average F1 score as the tuning criterion (43).

### 2.9 Model evaluation metrics

To comprehensively evaluate model performance, multiple classification metrics were employed. First, the overall classification accuracy (Accuracy), as well as precision, recall, and F1 score for each class, were calculated to assess model performance under class-imbalanced conditions (43, 44). Second, based on the predicted probabilities, the area under the receiver operating characteristic (ROC) curve (AUC) was computed for each class using a one-*vs*-rest (OvR) strategy. The macro-average AUC (macro-AUC) was further reported as an indicator of the overall discriminative performance (45). Finally, column-normalized confusion matrices were constructed to provide an intuitive visualization of the classification distribution across different categories (43, 44).

To further evaluate model generalizability and potential overfitting beyond the nested cross-validation framework, two additional analyses were conducted (1). Using the features and hyperparameters selected through cross-validation, a final version of each model was refitted on the full dataset. The apparent macro AUC and balanced accuracy were then calculated on the training set and compared with the performance obtained from outer-fold out-of-fold predictions. The difference (Δ = apparent − CV) was taken as an estimate of model optimism (2). The macro AUC and balanced accuracy obtained from the validation sets of each outer fold were treated as independent data-split results. Their mean, standard deviation, and 95% confidence intervals (CI) were computed to quantify the stability of performance estimates across different data partitions.

### 2.10 Model interpretation

To enhance model interpretability, the SHAP (SHapley Additive exPlanations) method was applied to quantify the contribution of each predictor (46). Specifically, within each outer validation fold of the nested cross-validation framework, SHAP values were computed for the predicted probability functions of the three outcome classes. For each feature, the mean of the absolute SHAP values was calculated across samples, followed by an arithmetic average across the three classes to obtain the macro-averaged |SHAP| for that fold. Finally, the mean of the macro-averaged |SHAP| values across all outer folds was computed to derive a robust, cross-fold ranking of global feature importance.

## 3 Results

### 3.1 Comparison of general and clinical characteristics across metabolic risk levels

Group differences in demographic and clinical characteristics among the three metabolic risk levels are summarized in **Table 1**. The distribution of sex differed significantly across groups (*χ²* = 8.504, *p* = 0.014). The MS group had the highest proportion of males (60.4%), whereas females were predominant in the High-risk group (64.7%). Age showed a marginally significant difference among the three groups (*H* = 6.010, *p* = 0.0495), while age at onset differed significantly (*H* = 10.792, *p* = 0.005). Post hoc Dunn–Holm tests indicated that the Critical group had a significantly later age at onset compared with the MS group. Although duration of illness exhibited a trend toward group differences (*H* = 5.188, *p* = 0.075), it did not reach statistical significance.

**Table 1:** Comparison of demographic and clinical characteristics across metabolic risk groups in patients with schizophrenia.

Variable		High risk group (*n* = 34)	Critical group (*n* = 88)	MS group (*n* = 91)	*X^2^/H*	*P-value*
Sex	Male	12 (35.3%)	38 (43.2%)	55 (60.4%)	8.504	0.014
	Female	22 (64.7%)	50 (56.8%)	36 (39.6%)		
Age		32.50 [28.00,35.00]	35.00 [29.00,39.00]	35.00 [28.50,37.50]	6.010	0.0495
Age at Onset		25.50 [21.20,30.80]^ab^	27.50 [24.00,31.00]^a^	23.00 [22.00,27.00]^b^	10.792	0.005
Duration		5.50 [3.25,8.75]	7.00 [5.00,11.00]	9.00 [4.00,12.00]	5.188	0.075
Education		12.00 [11.00,12.00]^ab^	12.00 [11.00,12.00]^a^	9.00 [9.00,12.00]^a^	30.900	<0.001
Marital Status	Unmarried, without partner	18 (52.9%)	36 (40.9%)	41 (45.1%)	/	0.261
	Unmarried, with partner	8 (23.5%)	25 (28.4%)	27 (29.7%)		
	Married	5 (14.7%)	26 (29.5%)	20 (22.0%)		
	Other	3 (8.8%)	1 (1.1%)	3 (3.3%)		
Living Arrangement	Living alone	8 (23.5%)	19 (21.6%)	30 (33.0%)	/	0.239
	Living with spouse	8 (23.5%)	26 (29.5%)	15 (16.5%)		
	Living with parents	16 (47.1%)	36 (40.9%)	43 (47.3%)		
	Other	2 (5.9%)	7 (8.0%)	3 (3.3%)		
Upbringing	Raised by both parents	16 (47.1%)	49 (55.7%)	47 (51.6%)	/	0.359
	Raised by single mother	11 (32.4%)	11 (12.5%)	16 (17.6%)		
	Raised by single father	2 (5.9%)	7 (8.0%)	6 (6.6%)		
	Left-behind or other caregiving situations	5 (14.7%)	21 (23.9%)	22 (24.2%)		
Smoking	No	21 (61.8%)	55 (62.5%)	40 (44.0%)	7.074	0.029
	YES	13 (38.2%)	33 (37.5%)	51 (56.0%)		
Alcohol	No	32 (94.1%)	80 (90.9%)	83 (91.2%)	/	0.896
	YES	2 (5.9%)	8 (9.1%)	8 (8.8%)		
Family History of Psychosis	No	29 (85.3%)	72 (81.8%)	63 (69.2%)	5.575	0.062
	YES	5 (14.7%)	16 (18.2%)	28 (30.8%)		
Family History Metabolic	No	32 (94.1%)	77 (87.5%)	76 (83.5%)	/	0.327
	YES	2 (5.9%)	11 (12.5%)	15 (16.5%)		
Antipsychotic Combination	No	25 (73.5%)	58 (65.9%)	50 (54.9%)	4.414	0.110
	YES	9 (26.5%)	30 (34.1%)	41 (45.1%)		
Antipsychotic Risk	Low risk	13 (38.2%)	19 (21.6%)	18 (19.8%)	9.915	0.042
	Medium risk	8 (23.5%)	31 (35.2%)	20 (22.0%)		
	High risk	13 (38.2%)	38 (43.2%)	53 (58.2%)		
P subscore		16.00 [14.00,18.00]	18.00 [15.00,20.20]	17.00 [15.00,19.00]	3.006	0.223
N subscore		16.00 [14.00,17.00]	16.00 [15.00,18.00]	17.00 [15.00,19.00]	3.515	0.173
G subscore		37.00 [33.20,38.00]	36.50 [34.00,41.00]	37.00 [34.00,40.00]	1.667	0.435
Total score		70.00 [64.20,73.80]	71.50 [66.00,77.00]	71.00 [68.00,76.00]	1.187	0.552

Categorical variables are presented as frequency (%) and compared using the Chi-square test.

Continuous variables are presented as median (25th percentile, 75th percentile).

Kruskal–Wallis test followed by Dunn–Holm post hoc test. Groups sharing the same letter do not differ significantly.

Detailed classification of Antipsychotic risk is provided in **Supplementary Table S1**.

Regarding other sociodemographic variables, there were no significant group differences in marital status (*p* = 0.261), living arrangement (*p* = 0.239), or upbringing environment (*p* = 0.359). A significant between-group difference was observed in smoking status (*χ²* = 7.074, *p* = 0.029), with the highest proportion of smokers found in the MS group (56.0%).

For medication-related variables, the antipsychotic risk category differed significantly across groups (*χ²* = 9.915, *p* = 0.042), with the MS group showing the highest proportion of patients receiving high-metabolic-risk antipsychotics (58.2%). Other medication-related factors—such as concomitant use of multiple antipsychotics (*p* = 0.110), family history of metabolic disorders (*p* = 0.327), and family history of psychosis (*p* = 0.062)—did not reach statistical significance. Moreover, the PANSS scores, including positive (P), negative (N), general psychopathology (G) subscales, and total score, showed no significant differences among the groups (all *p* > 0.05).

### 3.2 Comparisons of cognitive function across metabolic risk levels

The three metabolic risk groups exhibited differences across multiple domains of cognitive performance, as shown in **Table 2**. Among the six domains of the MATRICS Consensus Cognitive Battery (MCCB), three showed statistically significant group differences. Processing speed differed significantly across the three groups (*H* = 12.677, *p* = 0.002), with *post hoc* tests indicating that the MS group scored significantly lower than the High-risk group.

**Table 2:** Comparison of cognitive function scores across metabolic risk groups in patients with schizophrenia.

Variable	High risk group (*n* = 34)	Critical group (*n* = 88)	MS group (*n* = 91)	*H, P-value*
Processing speed	33.00 [27.25,42.50]^a^	31.00 [22.00,39.25]^ab^	28.00 [23.00,31.50]^b^	*H* = 12.677,*P* = 0.002
Attention vigilance	28.50 [28.00,40.00]	28.00 [25.75,38.00]	28.00 [25.50,34.00]	*H* = 1.158,*P* = 0.560
Working memory	18.50 [16.00,26.00]	21.00 [17.00,27.00]	19.00 [16.00,26.00]	*H* = 2.077,*P* = 0.354
Verbal learning	30.50 [25.25,35.75]^a^	28.00 [23.75,34.00]^a^	26.00 [17.00,29.00]^b^	*H* = 19.102,*P*<0.001
Visual learning	26.50 [24.50,30.75]	26.00 [22.00,32.25]	26.00 [22.50,28.00]	*H* = 2.539,*P* = 0.281
Reasoning problem solving	35.00 [32.00,39.75]^a^	32.00 [29.00,35.00]^b^	30.00 [26.00,35.00]^b^	*H* = 23.752,*P*<0.001

Values are presented as median (25th percentile, 75th percentile).

Dunn–Holm post hoc tests were used for pairwise comparisons.

Groups sharing the same superscript letter do not differ significantly.

The most pronounced differences were observed in verbal learning and reasoning and problem solving (*H* = 19.102, *p* < 0.001; *H* = 23.752, *p* < 0.001), respectively. In both domains, the MS group achieved the lowest scores, the High-risk group the highest, and the Critical group fell in between. All pairwise group comparisons were statistically significant.

No significant group differences were found in the remaining cognitive domains—attention/vigilance, working memory, and visual learning (all *p* > 0.05). However, the MS group showed slightly lower median scores across several domains compared with the High-risk group, suggesting relatively greater cognitive impairment in this subgroup.

### 3.3 Feature selection and multicollinearity diagnostics

**Figure 1:**
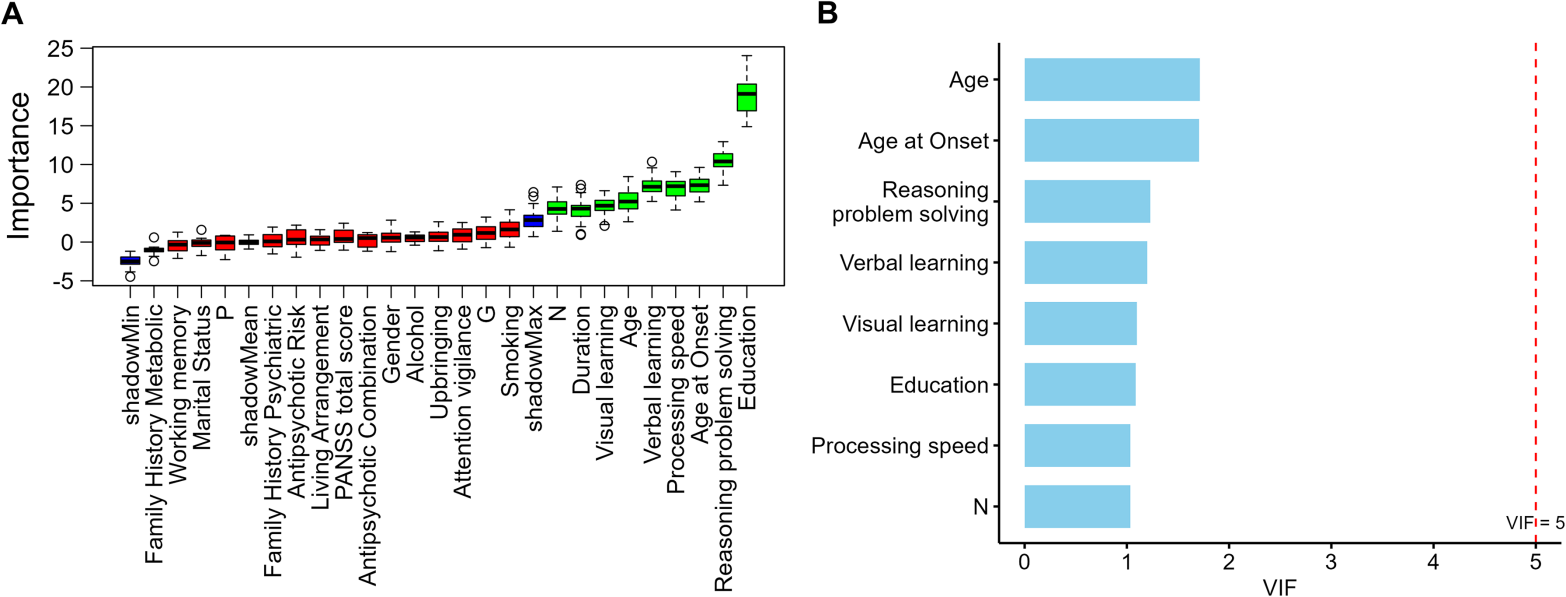
**(A)** Feature importance ranking by Boruta algorithm **(B)** Variance inflation factors of Boruta-selected variables.

To identify variables with predictive value for metabolic risk stratification in schizophrenia, the Boruta algorithm was first applied for feature selection, yielding nine candidate variables: Age, Age at Onset, Duration, Education, N score, Processing speed, Verbal learning, Visual learning, and Reasoning and problem solving. The results are presented in **Figure 1A**.

In the multicollinearity diagnostics, a linear dependency was detected between Duration, Age, and Age at Onset, with Duration showing a markedly higher initial variance inflation factor (VIF) compared with other variables. To ensure model stability, Age and Age at Onset were retained to represent the temporal dimension of illness course, while Duration was excluded. After re-evaluation, the remaining eight variables all had VIF values below 2 (range = 1.03–1.71; **Figure 1B**), indicating no significant multicollinearity.

A total of eight candidate predictors were ultimately retained for subsequent multinomial logistic regression and machine-learning analyses. Among these predictors, apart from age-related variables, most were years of education and cognitive-domain measures, with only the negative symptom score (PANSS N subscore) entering the analysis as a clinical symptom indicator. This pattern suggests that, when balancing overall model performance and stability, educational and cognitive characteristics were more consistently preserved as potential key predictors compared with clinical symptom variables.

### 3.4 Multinomial logistic regression analysis

To further examine the independent predictive role of cognitive function in metabolic risk stratification, a multinomial logistic regression analysis was performed using metabolic risk category as the dependent variable, with the High-risk group serving as the reference. The eight variables retained after feature selection were included in the model, and the results are summarized in **Table 3**.

**Table 3:** Multinomial logistic regression predicting metabolic risk group membership based on cognitive and demographic variables.

Variable	Critical *vs*. high-risk OR (95% CI)	*p*-value	MS *vs*. high-risk OR (95% CI)	*P*-value
Age	1.11(1.00,1.23)	0.057	1.09(0.98,1.22)	0.118
Age at Onset	0.99(0.88,1.11)	0.853	0.90(0.80,1.03)	0.116
Education	0.93(0.69,1.26)	0.651	0.61(0.44,0.83)	0.002
N subscore	1.02(0.93,1.12)	0.664	1.03(0.93,1.14)	0.558
Processing speed	0.94(0.90,0.99)	0.014	0.91(0.87,0.96)	<0.001
Verbal learning	0.96(0.90,1.03)	0.268	0.90(0.83,0.97)	0.005
Visual learning	1.02(0.96,1.08)	0.481	0.96(0.89,1.03)	0.236
Reasoning problem solving	0.80(0.72,0.90)	<0.001	0.80(0.71,0.90)	<0.001

In the Critical *vs*. High-risk comparison, reasoning and problem solving (OR = 0.80, 95% CI: 0.72–0.90, *p* < 0.001) and processing speed (OR = 0.94, 95% CI: 0.90–0.99, *p* = 0.014) emerged as significant negative predictors.

**Figure 2:**
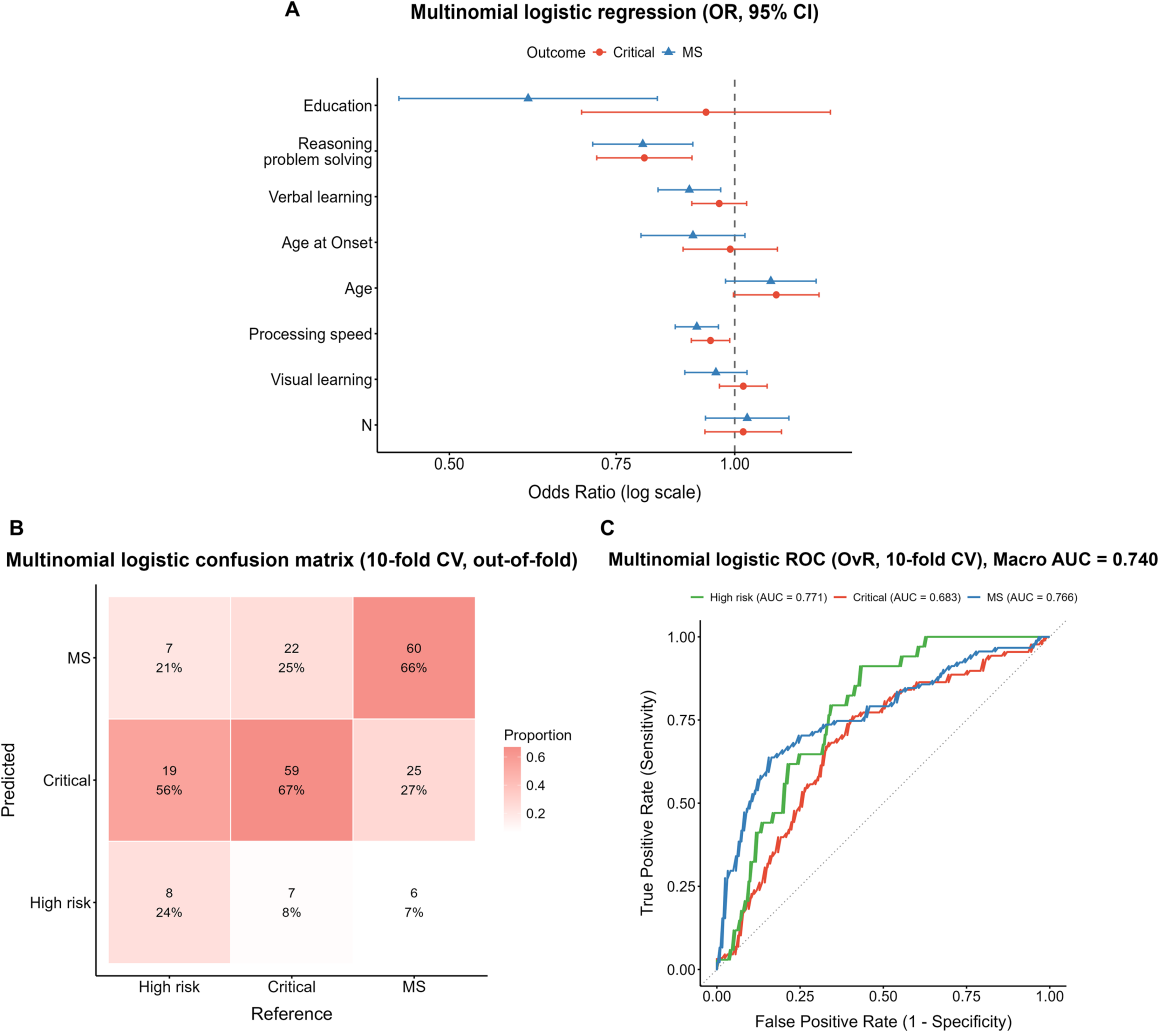
Performance of the multinomial logistic regression model. **(A)** Forest plot of odds ratios (ORs) with 95% confidence intervals, derived from the full-sample model. **(B)** Confusion matrix from 10-fold cross-validation (column-normalized, out-of-fold predictions). **(C)** One-vs-rest ROC curves with macro-average AUC from 10-fold cross-validation.

In the MS *vs*. High-risk comparison, reasoning and problem solving (OR = 0.80, 95% CI: 0.71–0.90, *p* < 0.001), processing speed (OR = 0.91, 95% CI: 0.87–0.96, *p* < 0.001), verbal learning (OR = 0.90, 95% CI: 0.83–0.97, *p* = 0.005), and years of education (OR = 0.61, 95% CI: 0.44–0.83, *p* = 0.002) were all significant negative predictors (**Figure 2A**).

The 10-fold cross-validation confusion matrix (**Figure 2B**) showed that among true High-risk cases, only 24% (8/34) were correctly identified, while 56% (19/34) were misclassified as Critical and 21% (7/34) as MS. In the Critical group, 67% (59/88) were correctly classified, 8% (7/88) were misclassified as High-risk, and 25% (22/88) as MS. In the MS group, 66% (60/91) were correctly identified, whereas 27% (25/91) were misclassified as Critical and 7% (6/91) as High-risk. ROC analysis (**Figure 2C**) yielded a macro-average AUC of 0.740. The AUCs for the High-risk, Critical, and MS subgroups were 0.771, 0.683, and 0.766, respectively.

Overall, in the multinomial logistic model that simultaneously included age, education, cognitive domains, and negative symptoms, the core cognitive domains—particularly processing speed and reasoning/problem solving—showed stable and independent predictive effects in differentiating metabolic risk levels, whereas negative symptoms did not demonstrate a corresponding independent contribution.

### 3.5 Random forest model

#### 3.5.1 Original dataset

**Figure 3:**
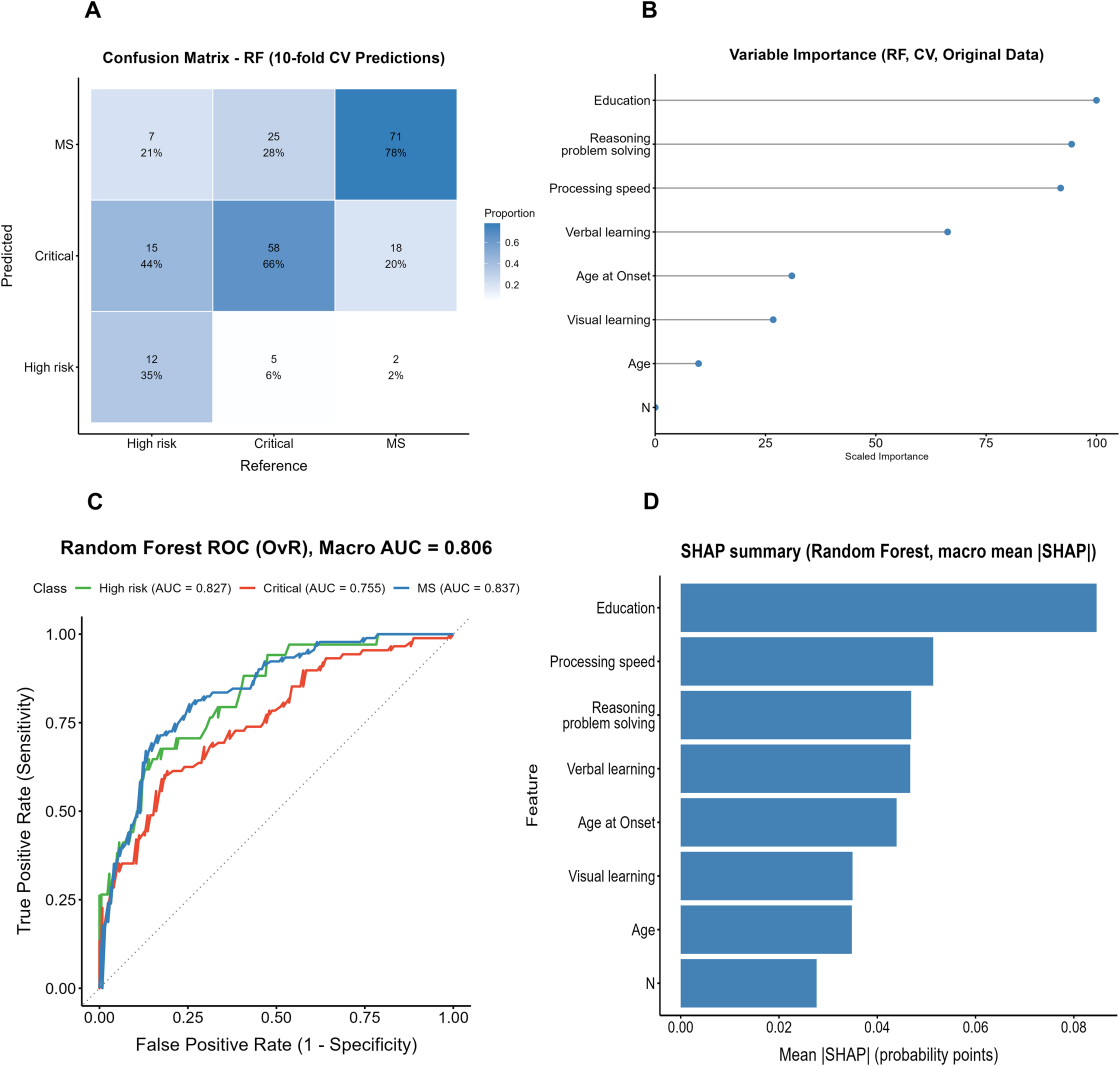
Performance and interpretability results of the Random Forest model (original data, cross-validation). **(A)** Confusion matrix of the Random Forest model. **(B)** Variable importance of the Random Forest model. **(C)** ROC curve of the Random Forest model (OvR, Macro AUC). **(D)** SHAP summary of the Random Forest model.

The confusion matrix of the random forest (RF) model constructed on the original dataset is shown in **Figure 3A**. Among the true High-risk cases, only 35% (12/34) were correctly identified, while 44% (15/34) were misclassified as Critical and 21% (7/34) as MS. In the Critical group, 66% (58/88) were correctly classified, whereas 28% (25/88) were misclassified as MS and 6% (5/88) as High-risk. The MS group showed the highest accuracy, with 78% (71/91) correctly identified, though 20% (18/91) were still misclassified as Critical.

ROC analysis (**Figure 3C**) yielded a macro-average AUC of 0.806, with AUCs of 0.827, 0.755, and 0.837 for the High-risk, Critical, and MS groups, respectively. Both the variable importance ranking and the SHAP analysis (**Figures 3B, D**) indicated that years of education contributed most to the model, followed by processing speed and reasoning/problem solving, whereas the clinical symptom indicator (PANSS N subscore) showed markedly lower relative importance.

#### 3.5.2 Random forest model on the SMOTE-oversampled dataset

**Figure 4:**
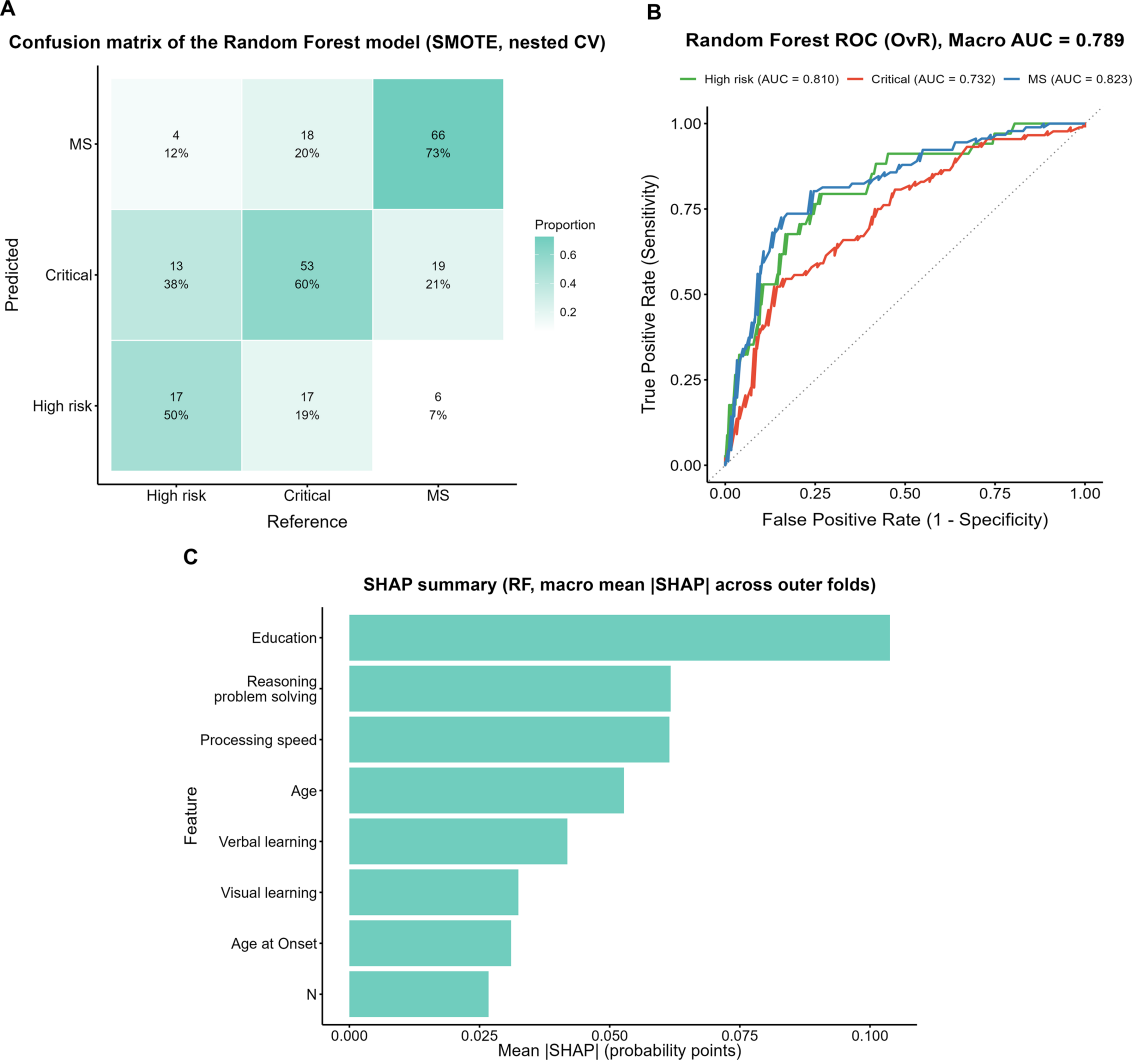
Performance evaluation and feature importance of the Random Forest model with SMOTE. **(A)** Confusion matrix of the Random Forest model with SMOTE (nested CV). **(B)** ROC curves of the Random Forest model with SMOTE (OvR). **(C)** SHAP summary plot (macro mean across outer folds).

The confusion matrix of the random forest (RF) model trained on the SMOTE-oversampled dataset is shown in **Figure 4A**. Among true High-risk cases, 50% (17/34) were correctly identified, whereas 38% (13/34) were misclassified as Critical and 12% (4/34) as MS. In the Critical group, 60% (53/88) were correctly classified, while 19% (17/88) were misclassified as High-risk and 20% (18/88) as MS. The MS group showed the highest accuracy, with 73% (66/91) correctly identified; however, 21% (19/91) were misclassified as Critical and 7% (6/91) as High-risk.

ROC analysis (**Figure 4B**) yielded a macro-average AUC of 0.789, with subgroup AUCs of 0.810, 0.732, and 0.823 for the High-risk, Critical, and MS groups, respectively. The SHAP analysis (**Figure 4C**) indicated that years of education contributed the most to the model, followed by reasoning and problem solving and processing speed.

### 3.6 XGBoost model

**Figure 5:**
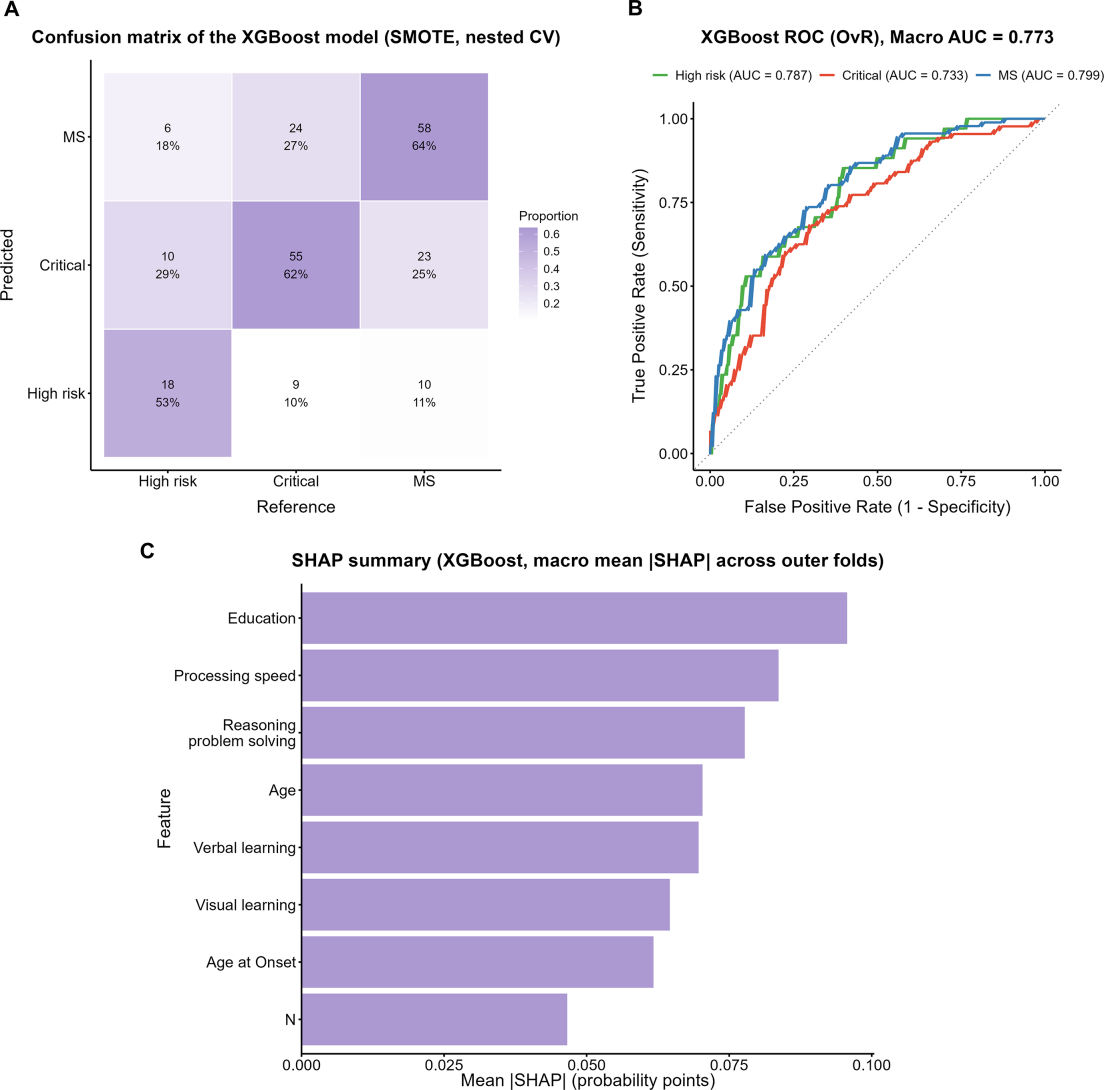
Performance evaluation of the XGBoost model with SMOTE. **(A)** Confusion matrix of the XGBoost model. **(B)** One-vs-Rest ROC curves with macro AUC. **(C)** SHAP summary plot showing macro mean ISHAPI across outer folds.

The confusion matrix of the XGBoost model trained on the SMOTE-oversampled dataset is shown in **Figure 5A**. Among true High-risk cases, 53% (18/34) were correctly identified, whereas 29% (10/34) were misclassified as Critical and 18% (6/34) as MS. In the Critical group, 62% (55/88) were correctly classified, while 10% (9/88) were misclassified as High-risk and 27% (24/88) as MS. In the MS group, 64% (58/91) were correctly identified, although 25% (23/91) were misclassified as Critical and 11% (10/91) as High-risk.

ROC analysis (**Figure 5B**) yielded a macro-average AUC of 0.773, with subgroup AUCs of 0.787, 0.733, and 0.799 for the High-risk, Critical, and MS groups, respectively. The SHAP analysis (**Figure 5C**) indicated that years of education contributed the most to the model, followed by reasoning and problem solving and processing speed.

### 3.7 SVM model

**Figure 6:**
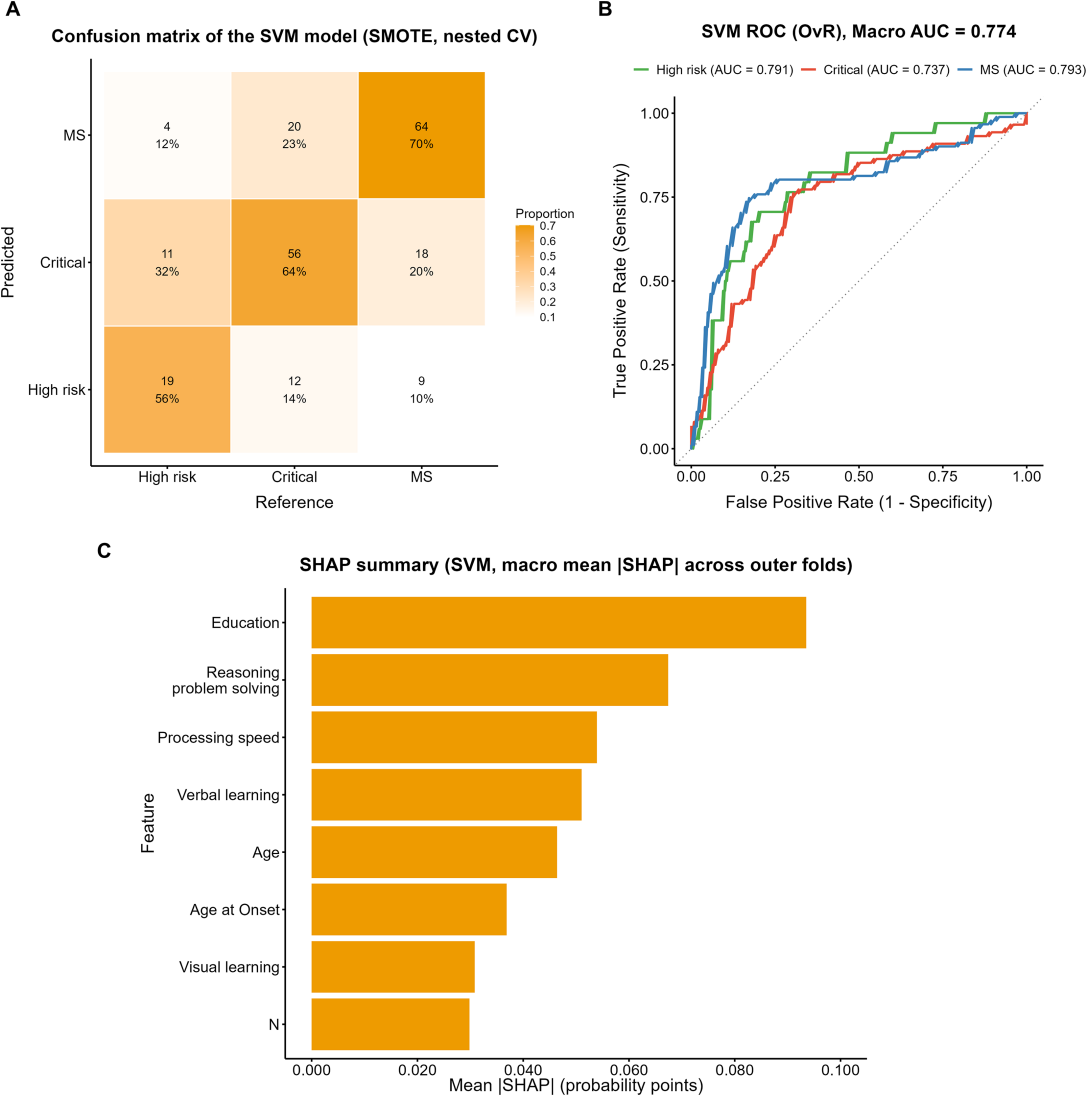
Performance evaluation of the SVM model with SMOTE. **(A)** Confusion matrix of the SVM model (SMOTE, nested CV). **(B)** ROC curves (OvR) of the SVM model with macro AUC. **(C)** SHAP summary plot showing macro mean ISHAPI across outer folds.

The confusion matrix of the SVM model trained on the SMOTE-oversampled dataset is shown in **Figure 6A**. Among true High-risk cases, 56% (19/34) were correctly identified, whereas 32% (11/34) were misclassified as Critical and 12% (4/34) as MS. In the Critical group, 64% (56/88) were correctly classified, while 14% (12/88) were misclassified as High-risk and 23% (20/88) as MS. The MS group showed the highest correct classification rate 70% (64/91), although 20% (18/91) were misclassified as Critical and 10% (9/91) as High-risk.

ROC analysis (**Figure 6B**) yielded a macro-average AUC of 0.774, with subgroup AUCs of 0.791, 0.737, and 0.793 for the High-risk, Critical, and MS groups, respectively. The SHAP analysis (**Figure 6C**) indicated that years of education contributed most to the model, followed by reasoning and problem solving, processing speed, and age, whereas verbal learning, visual learning, age at onset, and the PANSS N subscore showed comparatively lower contributions.

### 3.8 Model performance comparison and summary

Across the machine-learning models—including RF, XGBoost, and SVM—the SHAP analyses showed highly consistent patterns: years of education, reasoning and problem solving, and processing speed consistently ranked as the top three most important features, whereas clinical symptom indicators such as the PANSS N subscore exhibited notably lower relative importance.

**Figure 7:**
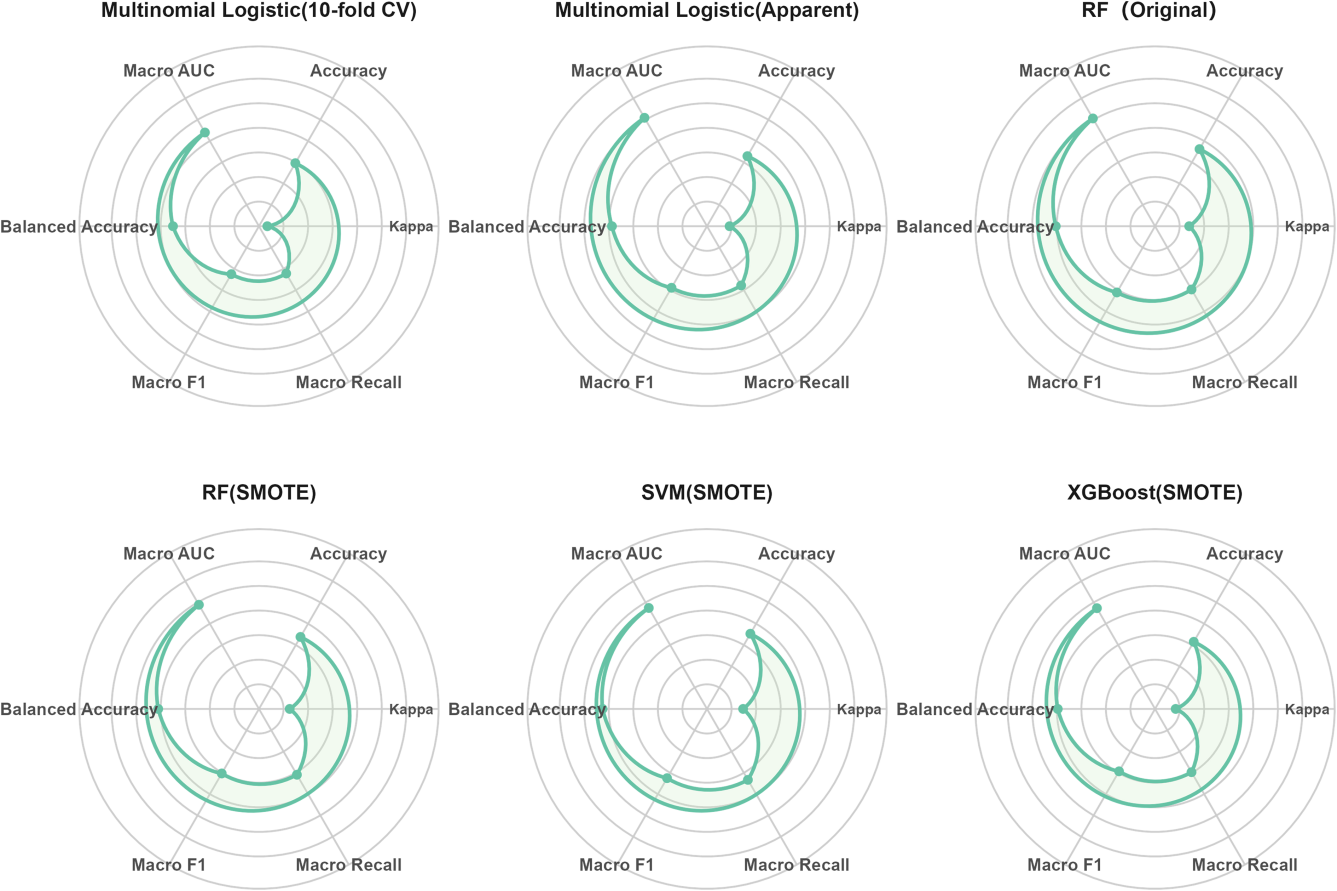
Visual comparison of multi-class metabolic risk prediction performance across models.

**Table 4** summarizes the performance of the multinomial logistic regression, RF, XGBoost, and SVM models in predicting metabolic risk stratification. To facilitate a visual comparison of overall performance across models, we additionally constructed a radar plot to summarize the main evaluation metrics (**Figure 7**). Overall, the machine-learning models outperformed the traditional multinomial logistic regression. Although the multinomial logistic regression model showed a relatively high apparent macro-average AUC in the full sample (0.809), its Kappa (0.392) and macro recall (0.578) were comparatively low; after 10-fold cross-validation, the overall performance further declined, suggesting that the apparent results may have been overly optimistic.

**Table 4:** Comparison of classification model performance metrics across different algorithms.

Model	Accuracy	Kappa	Macro recall	Macro F1	Balanced accuracy	Macro AUC
Multinomial Logistic (Apparent)	0.629	0.392	0.578	0.589	0.687	0.809
Multinomial Logistic (10-fold CV)	0.596	0.334	0.522	0.525	0.650	0.740
RF (Original)	0.662	0.439	0.597	0.611	0.704	0.806
RF (SMOTE)	0.638	0.425	0.609	0.603	0.710	0.789
XGBoost (SMOTE)	0.615	0.384	0.597	0.593	0.696	0.773
SVM (SMOTE)	0.653	0.447	0.633	0.625	0.725	0.774

The RF model trained on the original dataset achieved the best overall performance (accuracy = 0.662, macro F1 = 0.611, macro AUC = 0.806) and demonstrated stable results across multiple metrics. After applying SMOTE, both the macro recall and balanced accuracy of the RF model improved, indicating robustness in handling class imbalance. The SVM model with SMOTE achieved the highest macro recall (0.633), balanced accuracy (0.725), and macro F1 (0.625), highlighting its superior ability to identify minority classes. The XGBoost model with SMOTE showed intermediate performance between multinomial logistic regression and RF/SVM (accuracy = 0.615, macro AUC = 0.773); although not the best overall, it still outperformed the multinomial logistic regression baseline.

Stability and bias analyses (**Supplementary Tables S2, S3**) showed that the SVM model with SMOTE exhibited relatively small performance fluctuations across folds, with minimal differences between apparent and cross-validated performance. In contrast, the RF and XGBoost models showed higher apparent performance than cross-validated performance, suggesting a certain degree of optimism bias. Overall, the relative performance patterns observed in the stability and bias assessments were consistent with those in the main analyses, with the comparative strengths of the models remaining unchanged.

In summary, the RF and SVM models exhibited the best performance among all tested approaches, reflecting strengths in overall discrimination and minority-class recognition, respectively. The application of SMOTE further enhanced recall and class balance in most models, underscoring the potential value of machine-learning–based strategies for metabolic risk stratification in patients with schizophrenia.

## 4 Discussion

This study evaluated the predictive value of cognitive function, clinical symptoms, and general demographic and clinical information for metabolic risk stratification in patients with schizophrenia, and compared the predictive performance of traditional multinomial logistic regression with several machine-learning models. Overall, the findings indicate that years of education, reasoning and problem solving, processing speed, and other clinical/demographic variables have independent predictive value for identifying metabolic risk. Moreover, the machine-learning models exhibited superior discriminative performance compared with the traditional approach.

The main findings of this study fall into two areas. First, years of education, as well as the cognitive domains of processing speed, verbal learning, and reasoning/problem solving, were important predictors for distinguishing among the different levels of metabolic risk. This pattern highlights the central position of cognitive impairment in schizophrenia and indicates that cognitive deficits have a relatively independent contribution to the prediction of metabolic risk stratification. Second, in model comparisons, the random forest (RF) and support vector machine (SVM) yielded the strongest performance overall, reflecting complementary strengths in overall stability (RF) and minority-class recognition (SVM). Notably, the SVM showed an advantage in identifying the Critical group within an imbalanced dataset, capturing informative patterns that might be overlooked by conventional methods. These findings further support the potential application of machine-learning–based modeling strategies for metabolic risk stratification in patients with schizophrenia and point to possible directions for earlier clinical identification and intervention.

The present study identified processing speed, verbal learning, and reasoning and problem solving as effective predictors for differentiating metabolic risk levels, consistent with previous findings reporting significant associations between metabolic abnormalities and multidimensional cognitive impairments in schizophrenia (9, 47). Although some studies in early-stage schizophrenia failed to observe a clear link between metabolic dysregulation and cognitive deficits (13), the present results demonstrated that cognitive function retained independent predictive value even after controlling for clinical symptoms and demographic variables. This suggests that the association may persist across broader patient populations. Notably, our findings further highlighted the prominent role of specific cognitive domains—such as processing speed and reasoning/problem solving—in differentiating metabolic risk levels, aligning with hypotheses in the existing literature regarding potential associations between cognitive function and metabolic abnormalities in patients with schizophrenia (10, 48).

The association between cognitive function and metabolic risk may involve multiple biological processes, and this pattern of association appears to show some domain-specific characteristics across different cognitive dimensions. Chronic inflammatory responses have been shown to affect the prefrontal cortex and related neural circuits, thereby impairing executive function—including reasoning and problem solving—which may account for the strong contribution of this domain to metabolic risk differentiation (49). Insulin resistance and glucose dysregulation have been linked to reductions in white matter integrity, potentially impairing neural transmission efficiency and manifesting as slower information processing speed (50, 51). Moreover, disturbances in insulin signaling and hyperglycemia are associated with hippocampal dysfunction, which may directly weaken verbal learning capacity (50, 52). In addition, dyslipidemia and vascular risk factors may impair blood supply to the posterior cortical regions and associated perceptual pathways, adversely affecting visually mediated learning processes (51, 53, 54). Notably, this relationship may not be unidirectional: metabolic abnormalities and worsening cognitive impairment appear to be associated with each other, and cognitive deficits may also be related to elevated metabolic risk through their links with health behaviors and medication adherence. This possible bidirectional pattern of association suggests that cognitive indicators not only reflect disease status but also hold predictive relevance for metabolic risk levels and stratification.

Education also showed a stable predictive value, aligning with the view that schooling may confer protection against metabolic risk by enhancing cognitive reserve (55)and promoting healthier lifestyles and health behaviors (56). Our study further observed that years of education ranked among the top contributors across multiple models, suggesting that its role may extend beyond cognitive reserve to reflect broader socioeconomic conditions and capacity to utilize health resources (57). Within the context of metabolic risk prediction in schizophrenia, these findings provide a new lens through which to interpret the significance of educational factors.

In addition, age, age at onset, and negative symptom scores were retained during feature selection, indicating that illness course–related factors possess independent predictive value within a multivariable framework. Increasing age may reflect cumulative exposure to metabolic risk and diminishing physiological compensation, patterns that have been associated with stronger links between metabolic abnormalities and cognitive impairment (58, 59). Patients with earlier onset typically experience a longer duration of illness and more prolonged exposure to antipsychotic medication, often accompanied by impairments in social functioning; these factors may be associated with more severe cognitive deficits and an increased metabolic burden (60, 61). Negative symptoms have been suggested to influence physical activity, dietary management, and medication adherence through reduced motivation and social withdrawal, thereby increasing the risk related to metabolic abnormalities (62). Taken together, beyond educational and cognitive factors, age, illness duration, and symptom dimensions that reflect the course of illness are also significantly associated with metabolic risk stratification in patients with schizophrenia and make important contributions within the prediction models.

Moreover, the between-group differences in smoking (63), sex (64), and the metabolic risk category of prescribed antipsychotic medications (65)were consistent with prior epidemiological and clinical evidence. It is important to note that these factors are not only closely associated with metabolic health but may also be involved in processes related to cognitive impairment through pathways such as oxidative stress, vascular injury, or medication-related effects.

Methodologically, our findings showed that random forest and support vector machine outperformed traditional multinomial logistic regression in predictive performance, aligning with recent reports of superior machine-learning accuracy in psychiatric prediction tasks (66, 67).Unlike many studies that highlight a single algorithm, we observed complementary strengths: random forest provided the most stable overall discrimination, whereas SVM excelled in recognizing minority classes—particularly the Critical group. This pattern suggests that, for metabolic risk prediction in schizophrenia, model selection tailored to specific clinical needs and data characteristics may be more appropriate than reliance on any single method (68–70).

The present findings provide new insights into the early identification of metabolic risk among patients with schizophrenia. Traditional screening approaches primarily rely on biochemical indicators such as blood glucose and lipid levels. In contrast, this study demonstrated that cognitive function—particularly processing speed and reasoning ability—as well as years of education and other sociocognitive factors, possess independent predictive value in metabolic risk stratification. These results suggest that, beyond conventional metabolic monitoring, concise cognitive assessments could serve as a valuable complement for the early identification of high-risk individuals.

Furthermore, the close association between cognitive function and metabolic health implies that cognitive rehabilitation and metabolic management may have mutually reinforcing effects within intervention strategies. Prior studies have reported that improvements in cognitive function are often accompanied by reductions in metabolic risk levels, and that better metabolic control is frequently observed alongside enhanced cognitive performance (71, 72). Therefore, establishing an integrated multidisciplinary management model that encompasses psychiatry, endocrinology, and rehabilitation medicine could promote bidirectional improvements in metabolic and cognitive domains and ultimately enhance overall patient outcomes. The model developed in this study—based on brief clinical and cognitive indicators—is characterized by simplicity, low resource dependency, and practical applicability, suggesting strong potential for clinical implementation.

At the level of potential application, the multi-class prediction models developed from demographic and cognitive features may serve as clinical decision-support components embedded within hospital information systems or electronic medical records (73). Once years of education and key cognitive domains are routinely documented in a structured format, the information system could invoke the model in the background to generate individualized metabolic risk stratification results and provide clinicians with auxiliary prompts regarding metabolic monitoring frequency, antipsychotic medication selection, and lifestyle-related interventions (74). In community or outpatient follow-up settings, the core cognitive indicators identified in this study may also inform the development of simplified cognitive assessment scales or digital screening tools to facilitate the early identification of high-risk patients who may require further metabolic evaluation or intensified interventions (75). However, these application scenarios remain conceptual, and the clinical feasibility and effectiveness of the model will require further verification in prospective, multicenter studies.

This study has several limitations. First, the cross-sectional design restricts the ability to infer causal relationships among variables. Similarly, the SHAP-based model interpretation in this study can only depict the relative contributions of individual predictors to metabolic risk stratification, and the resulting findings should be understood as statistical associations at the model level rather than direct evidence of causal relationships or underlying pathophysiological mechanisms. Second, the sample size was relatively modest and derived from a single center, which may limit external validity. Third, we did not include detailed lifestyle information (e.g., diet, physical activity) or granular medication exposure data (e.g., drug class and dosage), which could have led to residual confounding and potential underestimation of their effects. Finally, although SMOTE was used to balance classes and enhance minority-class recognition, the risk of overfitting cannot be fully excluded and warrants evaluation in independent cohorts.

In addition, metabolic biomarkers and lifestyle-related factors were intentionally excluded from the candidate predictors to avoid overadjustment arising from high overlap between these variables and the diagnostic criteria for metabolic syndrome. This decision influenced the structural validity of the results to some extent: theoretically, the construct of metabolic risk is shaped jointly by metabolic indicators, lifestyle behaviors, and other upstream characteristics, whereas the model developed in this study identifies metabolic risk primarily based on demographic, clinical, and cognitive features. As such, it represents an early warning phenotype grounded in clinical and cognitive information rather than a full substitute for comprehensive assessments of metabolic biology and behavioral domains.

Future research may be further expanded in several directions. First, multicenter, large-sample, and longitudinal follow-up studies in independent samples from different regions and healthcare institutions are needed to conduct external validation and temporal external validation of the current model, in order to examine its discriminative performance, calibration, and stability across various service settings. Second, the model should be evaluated using broader clinical data and across patient groups at different illness stages to assess its robustness and generalizability in different subpopulations, as well as to compare its applicability across diverse clinical contexts. In addition, prospective implementation studies in real-world clinical settings are required, embedding the model as a clinical decision-support component within electronic medical records or follow-up systems to systematically evaluate its interpretability, acceptability, and its influence on metabolic monitoring behaviors and clinical outcomes. Such efforts would provide a practical foundation for precise risk assessment and individualized intervention for metabolic risk in patients with schizophrenia.

## Data Availability

The raw data supporting the conclusions of this article will be made available by the authors, without undue reservation.
