# Supplementary material for: The role of cognitive function in predicting metabolic risk in schizophrenia: a multi-model comparison incorporating clinical features
Source: Front Psychiatry. 2026 Jan 12;16:1724238. doi: 10.3389/fpsyt.2025.1724238 (PMC12832669; doi:10.3389/fpsyt.2025.1724238)
Supplement: Supplementary file 1 [file DataSheet1.docx]

***Supplementary Material***

***Supplementary Material 1. Metabolic Risk Classification of Antipsychotics***

This classification is derived from the Chinese Expert Consensus on the Management of Metabolic Syndrome in Patients with Schizophrenia (Chinese Journal of Psychiatry, 2020). The classification assigns antipsychotic medications to one of three risk levels: High, Medium, or Low, based on their impact on metabolic health. This table lists typical antipsychotics and their corresponding metabolic risk levels as outlined by the consensus.

Table S1. Metabolic risk classification of typical antipsychotics based on the Chinese expert consensus.

| Risk level | Typical antipsychotics |
| --- | --- |
| High | Clozapine, Olanzapine, Chlorpromazine |
| Medium | Quetiapine, Risperidone, Paliperidone |
| Low | Ziprasidone，Aripiprazole，Lurasidone，Amisulpride，Sulpiride，Perphenazine，Haloperidol |

Table note: This classification is derived from the Chinese Expert Consensus on the Management of Metabolic Syndrome in Patients with Schizophrenia (Chinese Journal of Psychiatry, 2020).

***Supplementary Material 2. Additional Robustness Checks for Model Performance***

Supplementary Tables S2 and S3 present the robustness analyses for the models used in the main analysis. The multinomial logistic regression is presented as a single model, with both apparent and cross-validated performance estimates listed together in Table S2. In the main manuscript, these estimates are presented separately for clarity. All other models (RF Original, RF SMOTE, XGBoost SMOTE, SVM SMOTE) are consistent with the main analysis and were evaluated in both apparent and cross-validation performance.

Table S2. Cross-validation fold-wise performance (mean, SD, and 95% CI).

| Model | Number of folds | Macro AUC, mean (SD) | Macro AUC, 95% CI | Balanced Accuracy  , mean (SD) | Balanced Accuracy  , 95% CI |
| --- | --- | --- | --- | --- | --- |
| Multinomial Logistic | 10 | 0.746（0.083） | 0.687-0.805 | 0.650（0.074） | 0.597-0.703 |
| RF  （Original） | 10 | 0.807（0.090） | 0.743-0.872 | 0.704（0.069） | 0.655-0.754 |
| RF  (SMOTE) | 5 | 0.796（0.068） | 0.712-0.881 | 0.710（0.054） | 0.643-0.777 |
| XGBoost  (SMOTE) | 5 | 0.783（0.041） | 0.732-0.833 | 0.696（0.040） | 0.647-0.746 |
| SVM  (SMOTE) | 5 | 0.783（0.079） | 0.687-0.883 | 0.725（0.066） | 0.643-0.808 |

Table note: The mean (mean), standard deviation (SD), and 95% confidence intervals (CI) of macro AUC and balanced accuracy were calculated based on the performance of each model across the outer folds of cross-validation. The 95% CI was estimated using t-distribution, reflecting the variability and stability of the model’s performance across different data splits.

Table S3. Apparent vs cross-validated performance and optimism (Δ).

| Model | Apparent Macro AUC | CV Macro AUC | Δ Macro AUC (App − CV) | Apparent Balanced accuracy | CV Balanced accuracy | Δ Balanced accuracy (App − CV) |
| --- | --- | --- | --- | --- | --- | --- |
| Multinomial Logistic | 0.809 | 0.740 | 0.069 | 0.687 | 0.650 | 0.037 |
| RF  （Original） | 1.000 | 0.806 | 0.194 | 1.000 | 0.704 | 0.296 |
| RF  (SMOTE) | 1.000 | 0.789 | 0.211 | 1.000 | 0.710 | 0.290 |
| XGBoost  (SMOTE) | 0.983 | 0.773 | 0.210 | 0.929 | 0.696 | 0.233 |
| SVM  (SMOTE) | 0.911 | 0.774 | 0.137 | 0.861 | 0.725 | 0.135 |

Table note: Apparent performance was obtained by refitting each model on the full dataset using the features and hyperparameters selected in nested cross-validation and evaluating performance on the same dataset. Cross-validated performance was obtained from pooled outer-fold predictions. Δ = Apparent − Cross-validated, representing the degree of model optimism (overfitting).
